# The UK Paediatric Familial Hypercholesterolaemia Register: Statin-related safety and 1-year growth data

**DOI:** 10.1016/j.jacl.2017.11.005

**Published:** 2018

**Authors:** Steve E. Humphries, Jackie Cooper, Peter Dale, Uma Ramaswami, FH Paediatric Register Steering Group

**Affiliations:** a Centre for Cardiovascular Genetics, Institute for Cardiovascular Science, University College London, London, UK; b Department of Paediatrics, Royal Gwent Hospital, Newport, Wales; c Lysosomal Disorders Unit, Royal Free Hospital, London, UK

**Keywords:** Familial hypercholesterolemia, Pediatric, LDL-C levels, Overweight, Obesity

## Abstract

**Background:**

For children with familial hypercholesterolemia (FH), UK guidelines recommend consideration of statin therapy by age 10 years and dietary and lifestyle advice to maintain an ideal body weight.

**Objectives:**

The objective of the study is to use the UK Paediatric Familial Hypercholesterolemia Register to determine: (1) the prevalence of plasma markers of liver toxicity and muscle damage in statin-treated FH children; (2) the prevalence of obesity in FH children compared to the UK general population; and (3) to compare growth rates in statin-treated and nontreated children.

**Methods:**

Differences in registration and 1-year characteristics were compared by Mann-Whitney *U* tests. Age and gender body mass index percentiles were compared to UK children's growth charts.

**Results:**

In 300 children (51% boys, 75% Caucasian, untreated mean [standard deviation] low-density lipoprotein cholesterol 5.50 [1.49] mmol/L), the proportion on statins varied significantly (*P* < .005) by age group (<5 years = 0%, 5–10 years = 16.7%, 10–15 years = 57.1%, and >15 years = 73.2%). Statin treatment reduced low-density lipoprotein cholesterol by 31% (1.84 [1.43] mmol/L), and no child showed elevated levels of markers of liver toxicity or muscle damage. At registration, 16.9% of the FH children were overweight (>85th percentile) and 11.1% were obese (>95th percentile) vs reported in 21.2% in UK non-FH children. There was no difference in annual growth rate in statin vs no-statin groups (age-adjusted weight increases 3.58 vs 3.53 kg; *P* = .91, height 4.45 vs 4.60 cm *P* = .73).

**Conclusions:**

We show no evidence for statin-related safety or growth issues, but many FH children over the age of 10 years are not on statin treatment. Fewer UK children with FH are obese compared to UK non-FH children.

## Introduction

Familial hypercholesterolemia (FH) is an autosomal dominant inherited disorder characterized by elevated low-density lipoprotein cholesterol (LDL-C) levels from birth,^1^ with premature coronary heart disease (CHD) occurring in roughly half of men by age 50 years and one-third of women by age 60 years.^2^ Statin therapy has been shown to significantly reduce CHD risk in FH patients.^2^ Although historically the prevalence of heterozygous FH (HeFH) is thought to be 1 in 500, recent studies have indicated the prevalence of HeFH in the United Kingdom and in countries in Europe may be twice as high.^3^, ^4^, ^5^ The underlying genetic cause for FH is most often due to mutations within the *LDLR* gene, which encodes the low-density lipoprotein receptor, but mutations in apolipoprotein B and proprotein convertase subtilisin/kexin type 9 can produce a phenotype identical to *LDLR* FH.^6^ In patients in whom no causative mutation can be found, a polygenic cause of their hyperlipidemia is most likely.^7^, ^8^ The detection of the causative mutation in a proband allows cost-effective DNA-based “cascade testing” in the family members, and this approach is recommended in most FH guidelines.^1^, ^9^, ^10^, ^11^, ^12^, ^13^ Once identified, the subjects with FH can be offered healthy lifestyle advice (eg, avoiding or stopping smoking) and lipid-lowering therapies.

All recent guidelines for the identification and management of adults and children with FH^1^, ^9^, ^10^, ^11^, ^12^, ^13^ have recommended the use of lipid-lowering statin therapy in children. In the United Kingdom, the 2008 NICE guideline (CG71) recommends that statin therapy should be considered by the age of 10 years. In the United Kingdom, atorvastatin is licensed in children over the age of 10 years up to a dose of 20 mg per day, whereas pravastatin is licensed from the age of 8 years with doses of 10–20 mg per day and in older children up to 40 mg daily.^14^ Recent European guidelines on the management of FH in childhood proposed that LDL-C be lowered below 3.5 mmol/L if possible.^13^ However, the age at which statin use should be started, or its intensity to best prevent the onset of adult premature CHD has not been rigorously established because there are no long-term randomized controlled outcome trials for ethical reasons. Encouragingly, a recent study indicated that initiating statin therapy in childhood resulted in fewer CHD events at a young age than had been seen historically in the parents.^15^ There is however considerable short-term randomized and observational data on the utility of statin therapy in children with HeFH, showing a good safety profile, without liver toxicity side effects, no influence on growth trajectory and excellent efficacy in terms of LDL-C reduction over periods of 2–3 years.^16^, ^17^ Where it has been examined, there have been no reports of significant increase in muscle pain and/or plasma levels of creatine kinase when on statins.

FH management guidelines, including the UK NICE guideline, recommend that all children (and adults) with HeFH should adopt healthy eating habits, be physically active, and make sensible lifestyle choices, to maintain an ideal body weight.^1^, ^9^, ^10^, ^11^, ^12^, ^13^ There is much current concern about the development of obesity in children, with the subsequent influence on morbidity, and a recent study of the Millennium children reported that between 11.8%–14.6% of 5- to 11-year-old UK children are overweight (body mass index [BMI] > 85th percentile) with 11.9%–21.2% being obese (BMI >95th percentile).^18^ We are unaware of any comparable data in UK FH children.^18^

The UK National Paediatric Familial Hypercholesterolaemia Register was established in 2012 to collect baseline and long-term follow-up data on all children with HeFH in the United Kingdom. We have previously published baseline data on a subset of these children, which demonstrated that treatment decisions in children with HeFH are appropriately based on a stronger family history of CHD and higher LDL-C.^19^ Here, we determined the prevalence of elevated levels of markers of liver toxicity and muscle damage in statin-treated children as an indicator of statin damage and examined the hypothesis that because of the dietary and lifestyle advice they receive, the prevalence of obesity in this cohort of FH children will be lower than that in the general population and that the growth rate from annual follow-up data will be similar in statin-treated and nontreated children.

## Methods

All lipid clinics in the United Kingdom and pediatricians with an interest in lipid disorders were contacted electronically and details of the register provided. An electronic web-based data capture tool was developed to collect information. The register captures routine clinical data, demography, family history, treatment, and lifestyle details, and clinicians are sent an electronic reminder to fill in annual follow-up data. Full details of the establishment and governance of the register have been published,^19^ as well as the data fields included in the electronic web-based data capture. UK ethical approval was obtained in November 2012 from the NRES Committee North East–Newcastle and North Tyneside (12/NE/0398). Written informed consent was obtained from the parents or guardians of the children who were mentioned on the register and, where appropriate, assent was obtained from the children. Patient information sheets, age-specific consents and assents, and parent information leaflets are available from the authors on request. Children were diagnosed as having FH based on the UK Simon Broome criteria,^2^ with the majority having been identified by family studies from an index case with a clinical diagnosis of FH. Of the children, 98.7% (232/235) have a diagnosis of HeFH, whereas 3 individuals had a total cholesterol >15 mmol/L compatible with a diagnosis of homozygous FH and were excluded from the analysis. For annual review, the most recent age, weight, height, plasma lipid profile, type and dose of statin if prescribed, and values of creatinine kinase (CK), alanine aminotransferase (ALT), and aspartate aminotransferase (AST) reported as part of routine clinical care were analyzed. Statin-induced toxicity was examined as the proportion of children having levels over 2.5 times the upper limit of normal as described in the Common Terminologies Criteria for Adverse Events guidelines.

### Statistical methods

Results for continuous variables are presented as mean (±standard deviation) and median (with interquartile range), and differences by sex and statin use are tested using Mann-Whitney *U* tests. The BMI was calculated as (mass in kg)/(height in m)^2^. Age- and gender-specific BMI percentiles were calculated using the childhood BMI batch calculator https://www.cdc.gov/healthyweight/xls/bmi_group_calculator_english.xls.

Only those with both recorded weight and height were included. Differences in the prevalence of overweight and obesity were examined using a 2 × 2 chi-squared test. Differences in BMI height, weight, and the fall in LDL-C by statin use are adjusted for age using analysis of covariance. Changes in BMI weight and height were calculated as change per 1-year increase in age and are based on those with at least 1 year of follow-up, whereas changes in lipid levels are the difference between the follow-up and registration. Categorical variables are presented as percentages and number and tested using chi-squared tests or Fisher's exact test. Changes in LDL-C by statin use were analyzed using analysis of covariance with adjustment for age and length of follow-up. For conversion to mg/dL, mmol/L levels of total and LDL-C should be multiplied by 38.7.

## Results

Baseline data shown in Table 1 include 300 HeFH children (51% boys, 75% Caucasian), with an untreated mean (SD) LDL-C of 5.50 (1.49) mmol/L. After a mean (SD) duration of follow-up of 2.7 (2.4) years, overall 52.5% of the children were on statins at follow-up, but this varied significantly (χ^2^ = 34.6 *P* < .005) by age group, being 0% (0/2) in those under 5 years, 16.7% (7/42) in those between 5 and 10 years, 57.1% (80/140) in those between 10 and 15 years, and 73.2% (41/56) in those over 15 years (Online Fig. 1). In children both below and above the age of 10 years, LDL-C was significantly higher prior to commencing statins than in the untreated cohorts, LDL-C of 5.88 (1.49) mmol/L vs 5.21 (1.42) mmol/L (*P* = .0004), respectively, and those on statins also had stronger evidence of a family history of early CHD (overall 43.7% vs 32.7%; *P* = .05). The proportion of children with a reported FH-causing mutation was not significantly different in treated and nontreated children (66% vs 68%; *P* = .77).

**Table 1:** Characteristics at diagnosis by statin use

	No statin use (N = 165)	Statin use (N = 135)	*P* value
Age, y			
Mean (SD); N	9.5 (3.5); 165	10.7 (3.2); 135	.001
Median (IQR)	9.5 (6.8–12.3)	10.8 (8.9–13.4)	
Sex			
% Male	51.5% (85/165)	50.4% (68/135)	.84∗
Ethnicity			
% Caucasian	77.6% (128/165)	71.9% (97/135)	.26∗
Smoking, % (N)	1.23% (2/162)	1.5% (2/132)	1.00
Mutation status			
% Yes (number)	67.8% (101/149)	66.1% (84/127)	.77∗
CHD in parent/first-degree relative			
% Yes	18.2% (30/165)	24.2% (32/132)	.20∗
CHD in any relative			
% Yes	32.7% (54/165)	43.7% (59/135)	.05∗
CHD onset age in relative			
Mean (SD); N	40.8 (15.4); 54	38.3 (14.7); 59	.38
Median (IQR)	41 (36–51)	40 (34–46)	
Weight (kg)			
Mean (SD); N	37.4 (17.4); 150	42.1 (18.1); 121	.02
Median (IQR)	33.8 (23.5–48.8)	40.5 (28.5–51.9)	
Height (m)			
Mean (SD); N	1.37 (0.22); 141	1.45 (0.19); 107	.004
Median (IQR)	1.37 (1.22–1.55)	1.48 (1.32–1.59)	
BMI (kg/m^2^)			
Mean (SD); N	1918.6 (4.2); 141	19.2 (4.3); 107	.09
Median (IQR)	17.0 (15.6–20.9)	18.6 (16.2–21.1)	
Cholesterol (mmol/L)			
Mean (SD); N	7.15 (1.47); 165	7.79 (1.49); 135	.0003†
Median (IQR)	7.1 (6.2–8.1)	7.7 (6.8–8.7)	
HDL-C (mmol/L)			
Mean (SD); N	1.41 (0.34); 154	1.38 (0.30); 127	.70
Median (IQR)	1.36 (1.2–1.6)	1.4 (1.2–1.6)	
Triglyceride (mmol/L)			
Mean (SD); N	1.02 (0.52); 150	1.06 (0.56); 125	.47
Median (IQR)	0.8 (0.7–1.3)	0.9 (0.7–1.3)	
LDL-C (mmol/L)			
Mean (SD); N	5.21 (1.42); 158	5.88 (1.49); 123	.0004‡
Median (IQR)	5.1 (4.2–6.1)	5.8 (4.8–6.8)	

CHD, coronary heart disease; BMI, body mass index; HDL-C, high-density lipoprotein cholesterol; LDL-C, low-density lipoprotein cholesterol; SD, standard deviation; IQR, interquartile range.

∗ Wilcoxon tests were used to compare continuous variables between statin treatment groups, whereas chi-squared tests were used for categorical variables.

† Age-adjusted *P* value = 7.5 × 10^−6^.

‡ Age-adjusted *P* value = 2.1 × 10^−6^.

As shown in Online Table 1, the commonly used statins were atorvastatin (49.2%; n = 63), pravastatin (27.3%, n = 35), simvastatin (21.1%; n = 27), and rosuvastatin (2.3%; n = 3). There was no significant difference in age at treatment (*P* = .46) in those on different statins (means [ranges]) for age at treatment: pravastatin 11.2 (6–15), atorvastatin 12.0 (7–18), and simvastatin 12.5 (6–17). A small proportion of patients were on resins (2.2%), and 1 patient was reported to be on ezetimibe. No patients were on fibrates, and the use of plant stanols was limited (1.6%, n = 2). As shown in Online Table 2, in those on statin treatment, LDL-C levels were reduced compared to the diagnostic values by 31% (mean = 1.84 (SD = 1.43) mmol/L), and this reduction was slightly greater in those with no detected mutation compared to those with a detected mutation (36% [mean = 2.26 {SD = 1.50}] vs 27% [mean = 1.55 {SD = 1.38}] mmol/L, *P* = .04). This appears to be due to the higher baseline levels in those with no detected mutation because mean on-treatment levels were similar in both groups (Online Fig. 2). In the treated group, 55.6% still had levels over the suggested target of 3.5 mmol/L.

Finally, we examined the characteristics of the children over the age of 10 years, which is the age by which the UK NICE FH guideline and the European consensus guidelines^13^ recommend that initiation of statin therapy should be considered. As shown in Table 2, in this group (n = 71), 35% had evidence of a family history of early CHD, with the mean age of onset of CHD in any relative of 44.8 years, and 62% of the children carried an FH-causing mutation.

**Table 2:** Characteristics at diagnosis in those over 10 years without statin use (n = 71)

Age (y)	
Mean (SD)	12.79 (1.84)
Median (IQR)	12.57 (10.9–14.1)
Sex	
% Male	47.9 (34/71)
Ethnicity	
% Caucasian	76.1 (54/71)
Smoking, % (N)	2.9% (2/69)
Mutation status, % (N)	61.7% (37)
CHD in parent/first-degree relative	
% Yes	18.3% (13/71)
CHD in any relative	
% Yes	35.2% (25/71)
CHD onset age in relative	
Mean (SD)	44.8 (11.5)
Median (IQR)	46 (20–64)
Weight (kg)	
Mean (SD)	51.8 (14.5)
Median (IQR)	49 (42–59.4)
Height (m)	
Mean (SD)	1.57 (0.12)
Median (IQR)	1.56 (1.49–1.63)
BMI (kg/m^2^)	
Mean (SD)	21.0 (4.4)
Median (IQR)	20.6 (17.8–23.7)
Cholesterol (mmol/L)	
Mean (SD)	6.72 (1.37)
Median (IQR)	6.6 (5.8–7.5)
HDL-C (mmol/L)	
Mean (SD)	1.44 (0.36)
Median (IQR)	1.40 (1.2–1.65)
Triglyceride (mmol/L)	
Mean (SD)	1.08 (0.54)
Median (IQR)	0.9 (0.7–1.3)
LDL-C (mmol/L)	
Mean (SD)	4.75 (1.31)
Median (IQR)	4.65 (3.85–5.5)
LDL-C >3.5 mmol/L, % (N)	82.3% (56)

CHD, coronary heart disease; BMI, body mass index; HDL-C, high-density lipoprotein cholesterol; LDL-C, low-density lipoprotein cholesterol; SD, standard deviation; IQR, interquartile range.

As shown in Online Figure 3, 82.3% (56/68 with diagnostic LDL-C recorded) had LDL-C over 3.5 mmol/L. In the 102 children over 10 years not on statins, no reasons were recorded for 20 children, but as shown in Online Table 3, in those giving a reason, 37.2% were because the clinician considered the risk was low, 31.4% were attending their first clinic visit, awaiting DNA or repeat lipid measures, and were trying dietary measures, and 14% were being started on a statin after the current clinic visit. In only 12.8% was the reason given that the parent or patient had declined, and only 2.3% were due to statin intolerance in the patient (or parent).

We addressed the issue of the safety of statin use by analyzing the measures of plasma CK, ALT, and AST at follow-up. None of those on statins had measured plasma levels of CK, ALT, or AST indicative of statin toxicity (ie, > 2.5 times the normal range (Online Table 2 and Online Fig. 4)). Muscle pain was not recorded routinely in the register. However, in our experience and anecdotally, muscle symptoms in children taking statins are rare, usually mild, and resolve over a short period of time after starting statins. We are not aware of any patients on the register having reported statin-induced rhabdomyolysis.

**Figure 1:**
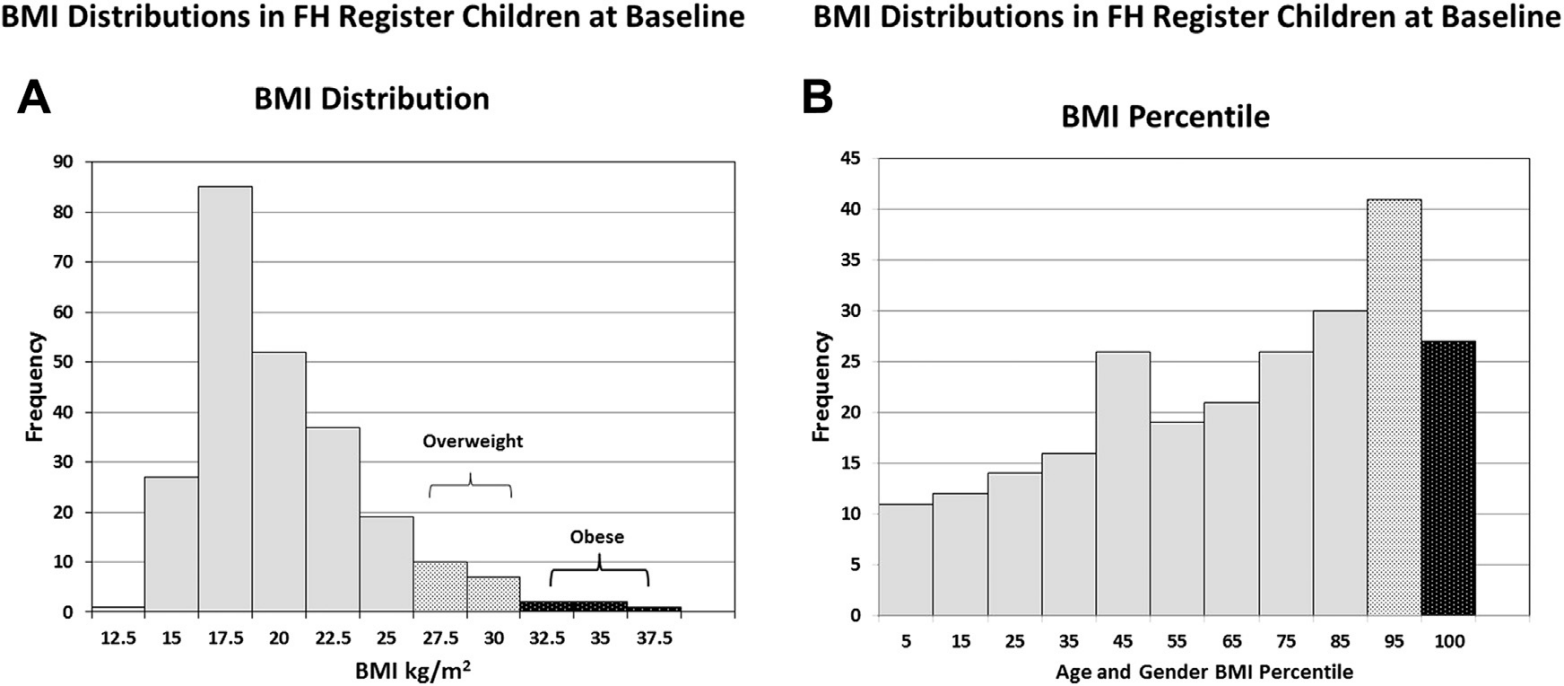
Distribution of (A) BMI and (B) age and gender BMI percentile in 243 FH children. (A) 17/243 = 7% had a BMI between 25 and 30 kg/m^2^ (light stipple bars) and 5/243 = 2.1% had a BMI > 30 kg/m^2^ (dark stipple bars). (B) 41/243 = 16.9% had an age- and gender-adjusted BMI percentile >85th percentile (light stipple bar) and 27/243 = 11.1% had an age- and gender-adjusted BMI percentile >95th percentile (dark stipple bar). BMI, body mass index; FH, familial hypercholesterolemia.

Online Figure 5A shows the baseline distribution of BMI by age, with the expected increase as children get older. As shown in Figure 1A, using BMI 25–30 kg/m^2^ as a measure of overweight and BMI >30 kg/m^2^ as obese, at diagnosis, 7.0% (17/243) would be classified as overweight and 2.1% (5/243) as obese. However, these BMI cutoffs are not appropriate for use in children.

The age- and gender-specific percentiles are used, with a BMI at or above the 85th percentile being designated as overweight and at or above the 95th percentile as obese.^18^ The age and gender percentile distribution is shown in Figure 1B, with 16.9% being overweight and 11.1% being obese. Compared to data from a large UK survey of non-FH children,^18^ the prevalence of overweight was similar (14.6% vs 16.9%; *P* = .33), but the prevalence of obesity was significantly lower (22.1% vs 11.1%; *P* = .0002).

In the annual review data (Online Fig. 5B), 17.6% had a BMI percentile indication of overweight and 11.9% as obese (not significantly different from the proportions seen at diagnosis). Changes in height and weight were calculated as change per 1-year increase in age and were based on those with at least 1 year of follow-up. As shown in Table 3, there was no significant difference in the increase in weight or height in those being statin treated compared with those not on statins (*P* = .91 and *P* = .73, respectively, after adjustment for age at diagnosis and sex).

**Table 3:** Change in height and weight by statin use

	No statin use	Statin use	*P* value
Change in weight (kg)			
Mean (SD); N	3.50 (2.29); 65	3.61 (2.52); 80	.97
Median (IQR)	3.39 (2.16–5.04)	3.34 (2.02–5.15)	
After adjustment for age at diagnosis and sex [mean (SD); N]	3.53 (2.42); 65	3.58 (2.41); 80	.91
Change in height (cm)			
Mean (SD); N	4.87 (2.63); 46	4.26 (2.39); 64	.14
Median (IQR)	5.27 (3.89–6.42)	4.49 (2.72–5.94)	
After adjustment for age at diagnosis and sex [mean (SD); N]	4.60 (2.17); 46	4.45 (2.16); 64	.73

Changes are calculated as change per 1-year increase in age and are based on those with at least 1 year of follow-up.

## Discussion

The main findings of this report are that, reassuringly, annual follow-up data showed no difference in average growth rate in the statin-treated children compared to the no-statin children, and none of those on statin had a clinically significant increase in measured plasma levels of CK, ALT, and AST, showing no evidence of any statin toxicity. From this perspective, our data are in keeping with the published short-term safety profile of statins in children (reviewed in the studies by Vuorio et al,^16^ Vuorio et al,^17^ and Emerson et al^20^). The long-term safety profile of statins in adults is well established.^21^, ^22^ The recent Dutch study reporting no CHD events in a 25-year follow-up of their treated children cohort, and evidence of greater longevity in the children than their FH parents,^15^ is supportive of the value of early statin therapy.

As a consequence of the elevated LDL-C seen in children with HeFH from birth, by the age of 10 years, they develop atherosclerosis, detectable as a significant degree of carotid intima-media thickness as compared with their non-FH siblings.^23^, ^24^ In a randomized controlled trial of the use of pravastatin, further increase in carotid intima-media thickness was prevented.^25^ Based on these data, the NICE guideline (CG71) and the European consensus guidelines^13^ state that the use of statins should be considered in children with HeFH by the age of 10 years using clinical judgment, based on the child's LDL-C level, age of onset of CHD in the parent or relatives, and presence of other CHD risk factors. The recent European expert opinion guideline^13^ suggested that in childhood, an on-treatment target LDL-C of 3.5 mmol/L would be ideal. Although there is no RCT or long-term follow-up of children to substantiate this target as actually having benefit in terms of CHD reduction, it is widely believed that such a therapy would prevent the development of significant atherosclerotic disease and subsequent cardiovascular events. In the register, there are 34 boys and 37 girls above the age of 10 years where statin therapy has not been initiated. Of these, over 80% have LDL-C over 3.5 mmol/L, and 35% have evidence of early CHD in a first-degree relative. As such, these children would be strong candidates for statin treatment. In the statin-treated children, LDL-C levels had been lowered by an average of 31% compared to the diagnostic level. However, 55.6% (75/135) still had levels over the suggested target of 3.5 mmol/L. From experience, we are aware that in some cases, this is due to poor adherence (eg, in adolescence), and the register does not specifically collect information on noncompliance. It could also be due to a clinical decision to postpone, as a parental, child, or clinician choice, the uptitration of statin dose for safety reasons until a child is older. It would be of interest to compare a noninvasive measure of subclinical atherosclerosis such as by determining carotid intima-media thickness in the treated and nontreated children in the register, but unfortunately, such measures are not routinely carried out in pediatric clinics and we have no data to address this.

The register collects data on reasons for not starting statin therapy, and this has allowed us to explore to what extent this could be due to reluctance from either clinicians or parents, for example, because of misconceptions on the longer term safety of statins when started in childhood. In 14% of cases, the records indicate that the child was being started on statin at the current clinic visit,^17^ whereas in 37% of cases, the recorded reason was because the clinician considered the child to be at low future risk, and in 31% being due to the child being registered at the first clinic visit, or with DNA testing or repeat lipid measures not having been completed. Follow-up studies will enable us to document what proportion of these children does indeed commence statin therapy. Only in 13% of children was this due to nonconsent from the parent and in less than 2% was this due to statin intolerance.

The second main finding is that the BMI distribution in these HeFH children identifies that a lower proportion of children are obese compared to the UK general population, whereas 16.9% of the HeFH children had a BMI suggestive of being overweight, which is not significantly different for the 14.6% reported in ∼14,000 non-FH UK children,^18^ only 11.1% were obese, which is roughly half the proportion seen in non-FH UK children, where 21.2% were obese by the age of 11 years. All FH guidelines recommend a healthy low-fat diet and exercise lifestyle as part of the management program for children (and adults) with FH, and it appears that this advice is being followed in the children seen here. None of the children have type I or type II diabetes (T2D). One of the potential long-term side effects of statin treatment in children with FH that needs to be considered is the higher risk of developing T2D that has been noted in statin treatment of non-FH patients. A meta-analysis of published RCTs in over 91,000 high-risk patients from the general population^26^ reported that statin therapy was associated with a 9% increase in the likelihood of new T2D during follow-up. Reassuringly, many studies have reported that the prevalence of T2D is low in adults with FH and in a study of over 63,000 subjects from Holland,^27^ even in treated adults, with FH the prevalence of T2D was significantly lower than in their unaffected relatives (1.75% vs 2.93%). Follow-up studies in adults^28^ and children^29^ are also reassuring, with a 10-year follow-up in 194 statin-treated children (mean age at baseline 13 years) seeing 1 new case of T2D, with a similar incidence in their 83 non-FH siblings.^29^ It would be of interest to examine whether there is any impact on glycemia in the treated children, but such tests are not routinely carried out in pediatric clinics and we have no data to address this.

The main strength of the register data is that it is a representative sample of children with FH who are being treated in pediatric and adult lipid clinics in the United Kingdom. Overall 67% of the children have a documented FH-causing mutation and have mostly been identified by cascade testing from an affected parent. Although some parents may not have consented to a genetic test, the most common reason for such tests not being carried out in children is due to the poor availability of funding of such tests in many parts of the country. The characteristics of the children are similar to that observed in the 2010 UK national audit of FH patients where notes on 147 children were examined (https://www.rcplondon.ac.uk/projects/outputs/familial-hypercholesterolaemia-audit). The vast majority of the children on the register have been identified by family studies from an index case with early CHD or elevated cholesterol levels, and as far as we are aware, none have been identified through lipid testing during childhood; they are therefore likely to be more severely affected than children detected, for example, by a national screening program.^4^ However, based on the age distribution in the United Kingdom and a prevalence of ∼1/250, there may be up to 56,000 children under the age of 18 years in the United Kingdom with FH, and it is clear that the majority of children with FH have yet to be diagnosed.

In conclusion, this longitudinal analysis of children on the UK FH register shows that the statin use in children is not associated with any reductions in growth rate and is safe in childhood, with no biochemical evidence of toxicity over a 2- to 3-year period. There appears therefore to be no contraindication to use statin lipid-lowering therapy in children with FH, who are very likely to show a significant long-term benefit in reduction in risk of future CHD.

## Supplementary data

Online Figure 1 Plot of the proportion of children on statin therapy by age group at follow-up. The number of children in each age group is 2, 42, 140, and 56, respectively.

Online Figure 2 Bar chart of the mean (SE) of LDL-C in the children at baseline in those who later were or were not on a statin and the fall in LDL-C seen upon statin treatment. Blue bar indicates baseline levels in those later not on statin; orange bar indicates baseline levels in those later on statin; and green bar indicates follow-up levels. The mean baseline levels in the children who were later put on a statin are nonsignificantly higher than in those not on treatment at follow-up, in both those with and without a documented mutation. LDL-C, low-density lipoprotein cholesterol.

Online Figure 3 Histogram of the distribution of diagnostic LDL-C in the children over the age of 10 years not on statin treatment. About 56/68 with diagnostic LDL-C recorded (82.3%) children have LDL-C > 3.5 mmol/L. LDL-C, low-density lipoprotein cholesterol

Online Figure 4 Histogram of the distribution of plasma markers of statin toxicity at follow-up. (A) alanine aminotransferase (ALT); (B) aspartate aminotransferase (AST); (C) creatine kinase (CK).

Online Figure 5 Scatter plot of the diagnostic BMI (A) and follow-up BMI (B). BMI, body mass index.
