# Supplementary figures and images for: The UK Paediatric Familial Hypercholesterolaemia Register: Statin-related safety and 1-year growth data
Source: J Clin Lipidol. 2018 Jan-Feb;12(1):25–32. doi: 10.1016/j.jacl.2017.11.005 (PMC5821682; doi:10.1016/j.jacl.2017.11.005)

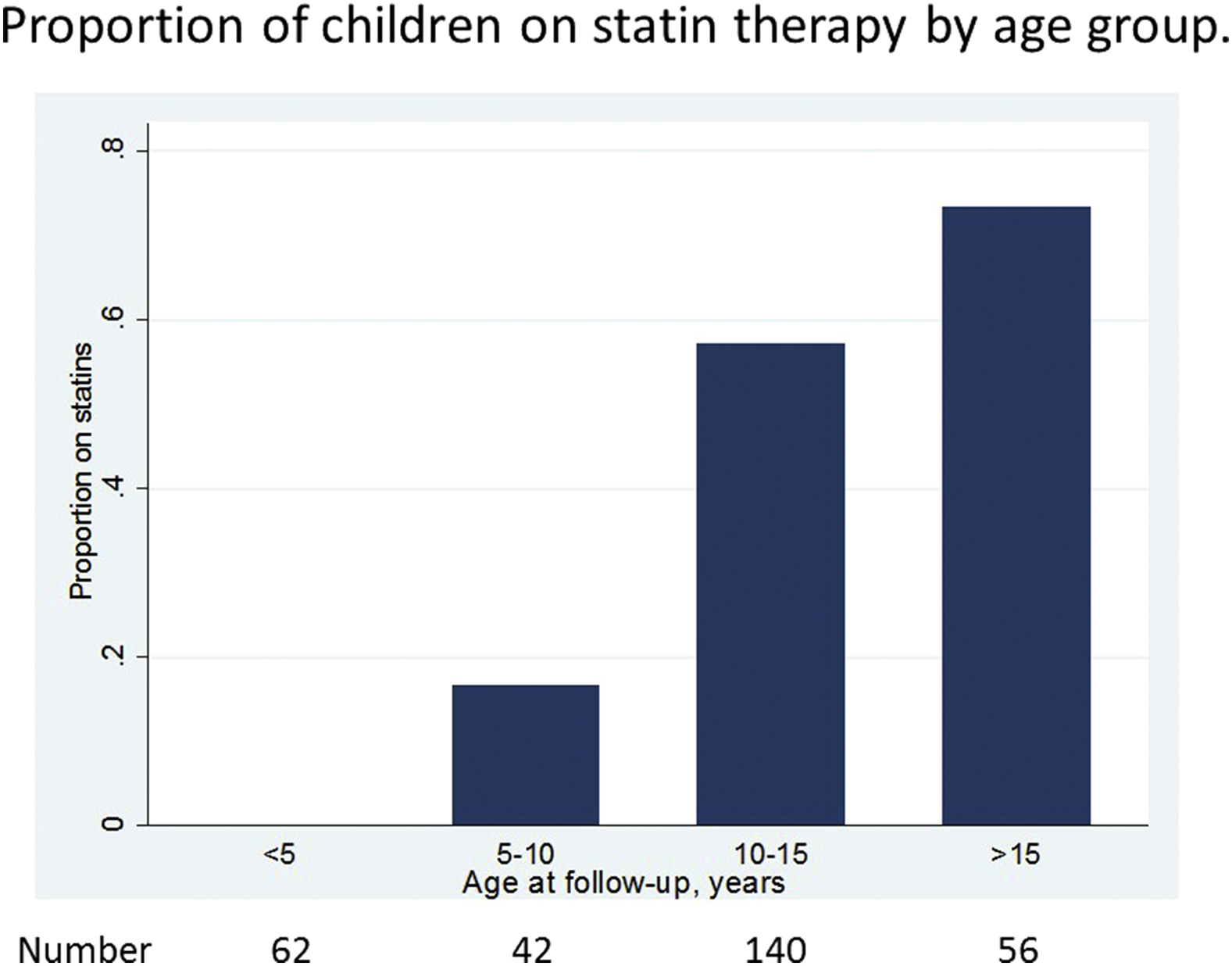

Supplement: Online Figure 1 — Plot of the proportion of children on statin therapy by age group at follow-up. The number of children in each age group is 2, 42, 140, and 56, respectively. [file figs1.jpg]

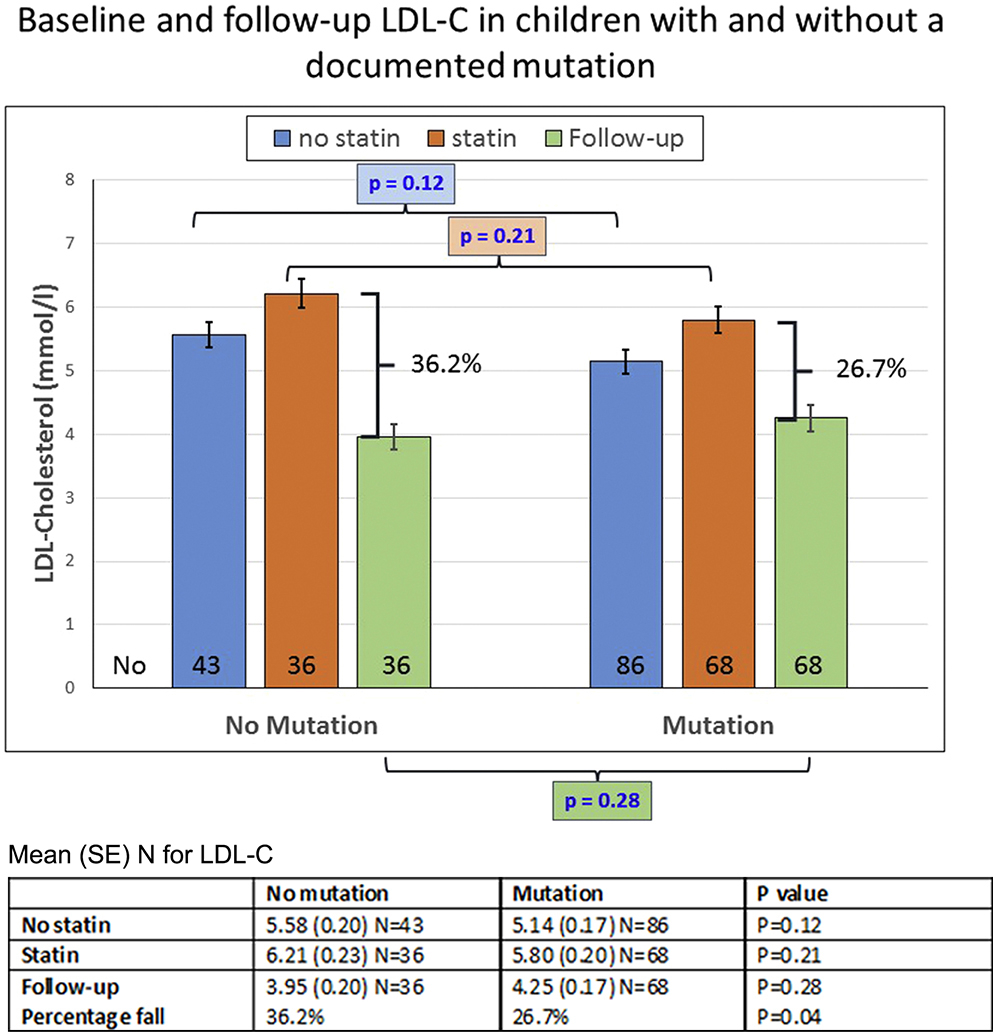

Supplement: Online Figure 2 — Bar chart of the mean (SE) of LDL-C in the children at baseline in those who later were or were not on a statin and the fall in LDL-C seen upon statin treatment. Blue bar indicates baseline levels in those later not on statin; orange bar indicates baseline levels in those later on statin; and green bar indicates follow-up levels. The mean baseline levels in the children who were later put on a statin are nonsignificantly higher than in those not on treatment at follow-up, in both those with and without a documented mutation. LDL-C, low-density lipoprotein cholesterol. [file figs2.jpg]

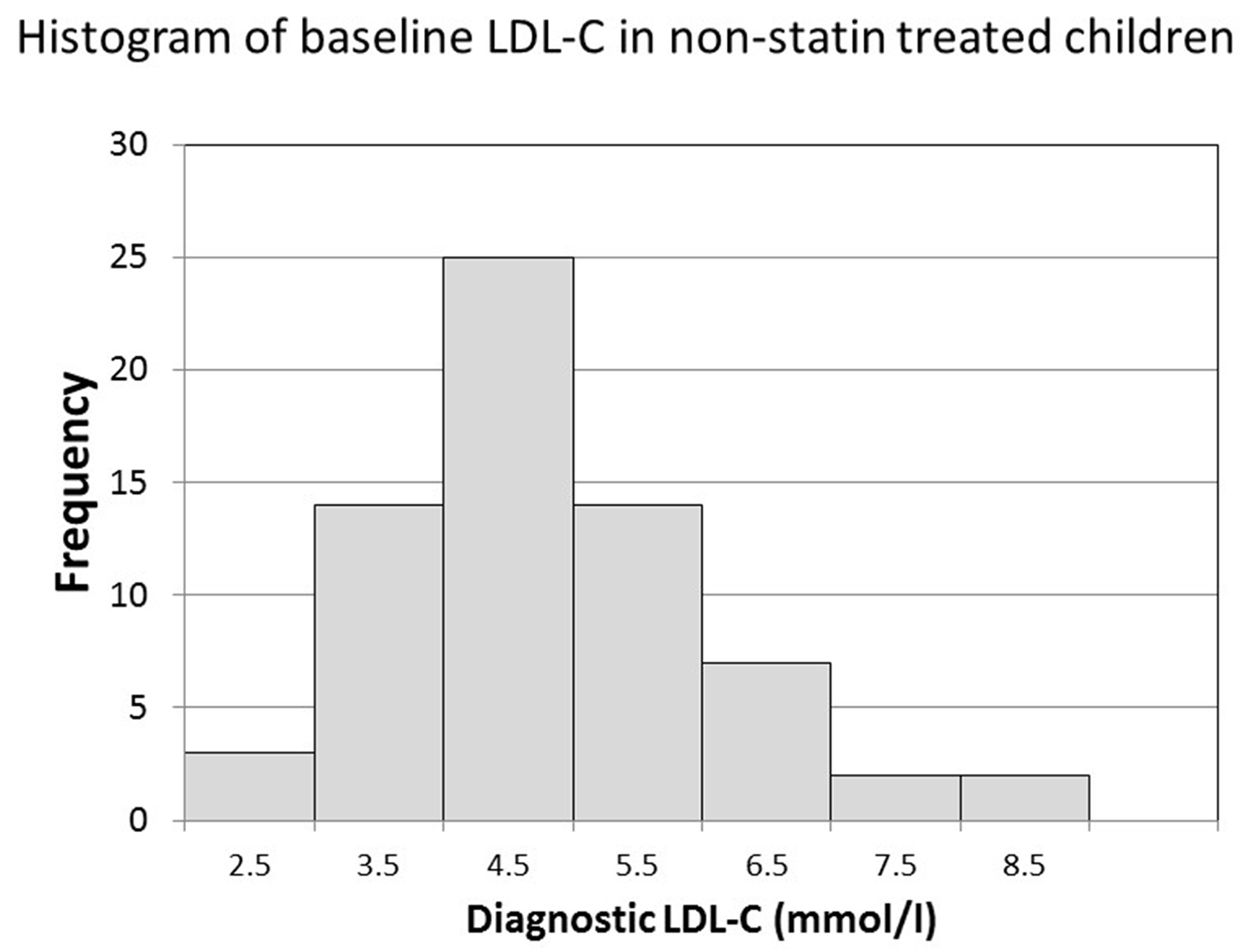

Supplement: Online Figure 3 — Histogram of the distribution of diagnostic LDL-C in the children over the age of 10 years not on statin treatment. About 56/68 with diagnostic LDL-C recorded (82.3%) children have LDL-C > 3.5 mmol/L. LDL-C, low-density lipoprotein cholesterol [file figs3.jpg]

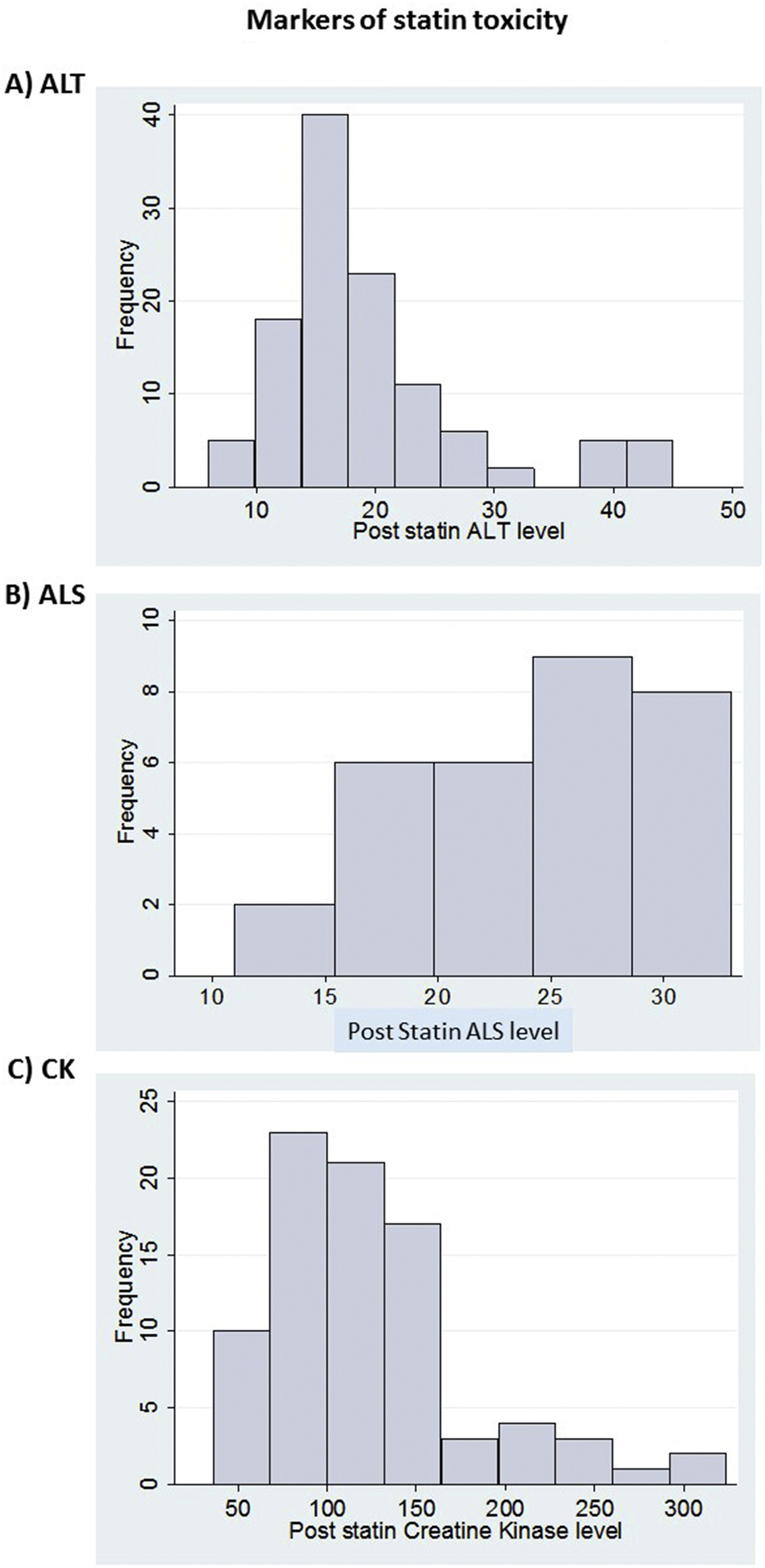

Supplement: Online Figure 4 — Histogram of the distribution of plasma markers of statin toxicity at follow-up. (A) alanine aminotransferase (ALT); (B) aspartate aminotransferase (AST); (C) creatine kinase (CK). [file figs4.jpg]

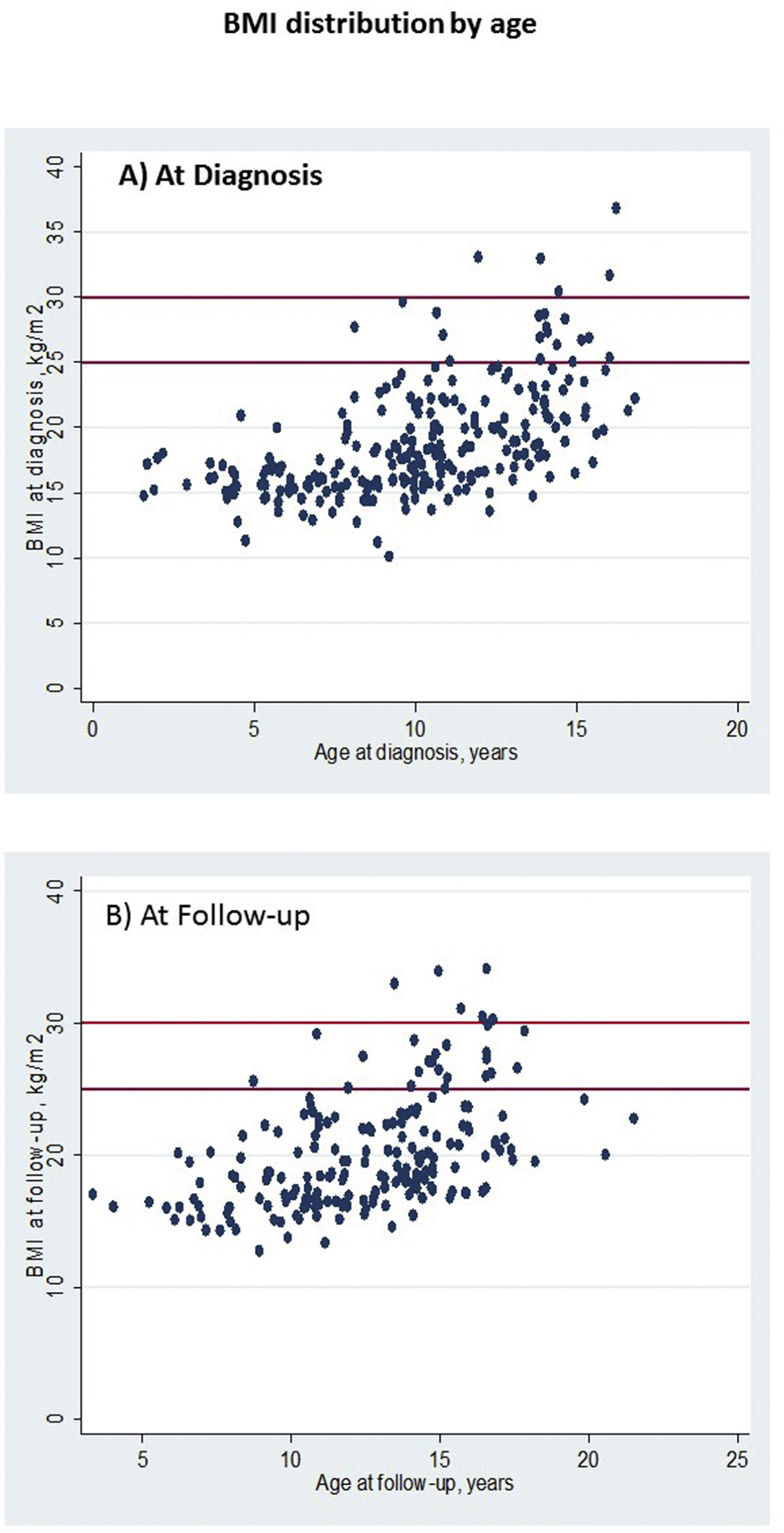

Supplement: Online Figure 5 — Scatter plot of the diagnostic BMI (A) and follow-up BMI (B). BMI, body mass index. [file figs5.jpg]
