# Supplementary material for: The UK Paediatric Familial Hypercholesterolaemia Register: Statin-related safety and 1-year growth data
Source: J Clin Lipidol. 2018 Jan-Feb;12(1):25–32. doi: 10.1016/j.jacl.2017.11.005 (PMC5821682; doi:10.1016/j.jacl.2017.11.005)
Supplement: Online Tables 1–3 [file mmc1.docx]

**Online Table1.** **Lipid Lowering treatment in Children with FH**

| **Drug** | **% on drug (N)** | **Dose** | **% on dose(N)** |
| --- | --- | --- | --- |
| Statin | 52.5% (128) | 5  10  20  30  40 | 3.9% (5)  64.8% (83)  25.8% (33)  1.6% (2)  3.9% (5) |
|  | Atorvastatin  49.2% (63)  Pravastatin 27.3% (35)  Rosuvastatin  2.3%  (3)  Simvastatin 21.1% (27) |  |  |
| Resin-Cholestyramine | 2.3% (3) | 4  8  12 | 33.3% (1)  33.3% (1)  33.3% (1) |
| Ezetimibe | 0.8% (1) | 10 | 100% (1) |
| Fenofibrate | 0 (0) | - | - |
| Other (Benecol, plant stannol) | 1.6% (2) | - | - |

**On line Table 2 Characteristics at last clinic appointment by statin use**

|  |  | No  N=116 | Yes  N=128 | P value  (Mann-Whitney) |
| --- | --- | --- | --- | --- |
| Age (years) | Mean (SD) N  Median [IQR] | 11.5 (3.3) 112  11.5 [9.3-14.2] | 13.9 (2.8) 128  14.2 (11.8-15.9) | 0.0001 |
| Weight (kg) | Mean (SD) N  Median [IQR] | 44.9 (18.6) 105  42 [29.9-55.4] | 53.4 (16.9) 123  52.8 [41.4-62.2] | 0.0002 |
| Height (m) | Mean (SD) N  Median [IQR] | 1.45 (0.19) 87  1.44 [1.34-1.60] | 1.57 (0.15) 108  1.59 [1.49-1.68] | 0.0001 |
| Cholesterol (mmol/L) | Mean (SD) N  Median [IQR] | 6.80 (1.33) 107  6.5 [5.9-7.8] | 5.73 (1.30) 126  5.7 [4.8-6.3] | 0.0001 |
| HDL-C (mmol/L) | Mean (SD) N  Median [IQR] | 1.42 (0.31) 101  1.4 [1.2-1.6] | 1.39 (0.30) 117  1.37 [1.2-1.5] | 0.27 |
| Triglyceride (mmol/L) | Mean (SD) N  Median [IQR] | 0.99 (0.51) 101  0.8 [0.64-1.23] | 0.94 (0.41) 116  0.84 [0.6-1.2] | 0.75 |
| LDL-C (mmol/L) | Mean (SD) N  Median [IQR] | 5.00 (1.27) 100  4.8 [4.0-5.9] | 3.98 (1.33) 114  3.9 [3.0-4.7] | 0.0001 |
| Fall in LDL-C from baseline (mmol/L) |  | 0.22 (0.75) N=145 4.2%  P= 0.0005 | 1.84 (1.43)  N=111 31%  P=3.7 x 10^-25^ | 0.0001 |

Differences in height and weight at latest clinic appointment are explained by age (age adjusted p=0.33 weight, p=0.78 height). Differences in cholesterol (p=0.00001), LDL-C (p=0.0003) and fall in LDL-C (p= 7.4 x 10^-21^) are independent of Age.

**On line table 3. Reasons given for those over 10 years not on statins (n=102)**

| **Reason Given** | **Number (percentage*)** |
| --- | --- |
| No reason given | 20 |
| Patient or parent intolerant to statin | 2 (2.3%) |
| Patient/parent /carer declined | 11 (12.8%) |
| Child over 10 years but risk low | 32 (37.2%) |
| Waiting for DNA test or repeat lipid measures | 12 (14.0%) |
| Starting statin after current clinic visit | 12 (14.0%) |
| First clinic visit and trying lifestyle changes | 15 (17.4%) |

*Four records gave more than one reason so percentages given out of 86 records where reason given

**Online Table 3 Markers of statin damage by statin use**

| **Creatinine Kinase** |  |  | P value (Mann-Whitney) |
| --- | --- | --- | --- |
| Pre statin | Mean (SD)  Median [IQR | 115.7 (48.4) 85  103 [83-137] | 0.73 |
| Post statin | Mean (SD)  Median [IQR | 121.4 (57.8) 83  104 [83-147] | 0.28 |
| Change | Mean (95% CI) | 7.6 (-2.33 to 17.63) N=65 |  |
|  | Signrank test | P value P=0.39 |  |
| **ALT** |  |  |  |
| Pre statin | Mean (SD)  Median [IQR | 17.4 (7.7) 102  15 [12-19] | 0.38 |
| Post statin | Mean (SD)  Median [IQR | 19.3 (8.6) 114  17 (14-22) | 0.21 |
| Change | Mean (95% CI) | 1.61 (0.10 to 43.11) N=97 |  |
|  | Signrank test | P value P=0.008 |  |
| **AST** |  |  |  |
| Pre statin | Mean (SD)  Median [IQR | 24.1 (7.5) 33  24 [18-27] | 0.51 |
| Post statin | Mean (SD)  Median [IQR | 24.1 (6.0) 31  25 [19-29] | 0.92 |
| Change | Mean (95% CI) | 0.52 (-1.85 to 2.89) N=25 |  |
|  | Signrank test | P value P=0.26 |  |
